# Knowledge-Based Reconstruction of mRNA Transcripts with Short Sequencing Reads for Transcriptome Research

**DOI:** 10.1371/journal.pone.0031440

**Published:** 2012-02-01

**Authors:** Junhee Seok, Weihong Xu, Hui Jiang, Ronald W. Davis, Wenzhong Xiao

**Affiliations:** 1 Stanford Genome Technology Center, Palo Alto, California, United States of America; 2 Department of Statistics, Stanford University, Stanford, California, United States of America; 3 Department of Biostatistics, University of Michigan, Ann Arbor, Michigan, United States of America; 4 Massachusetts General Hospital, Harvard University, Boston, Massachusetts, United States of America; Kyushu Institute of Technology, Japan

## Abstract

While most transcriptome analyses in high-throughput clinical studies focus on gene level expression, the existence of alternative isoforms of gene transcripts is a major source of the diversity in the biological functionalities of the human genome. It is, therefore, essential to annotate isoforms of gene transcripts for genome-wide transcriptome studies. Recently developed mRNA sequencing technology presents an unprecedented opportunity to discover new forms of transcripts, and at the same time brings bioinformatic challenges due to its short read length and incomplete coverage for the transcripts. In this work, we proposed a computational approach to reconstruct new mRNA transcripts from short sequencing reads with reference information of known transcripts in existing databases. The prior knowledge helped to define exon boundaries and fill in the transcript regions not covered by sequencing data. This approach was demonstrated using a deep sequencing data set of human muscle tissue with transcript annotations in RefSeq as prior knowledge. We identified 2,973 junctions, 7,471 exons, and 7,571 transcripts not previously annotated in RefSeq. 73% of these new transcripts found supports from UCSC Known Genes, Ensembl or EST transcript annotations. In addition, the reconstructed transcripts were much longer than those from *de novo* approaches that assume no prior knowledge. These previously un-annotated transcripts can be integrated with known transcript annotations to improve both the design of microarrays and the follow-up analyses of isoform expression. The overall results demonstrated that incorporating transcript annotations from genomic databases significantly helps the reconstruction of novel transcripts from short sequencing reads for transcriptome research.

## Introduction

In large-scale clinical studies, most existing data sets focus on gene expression profiles; however, human transcriptome is undoubtedly more complex. While human genome encodes only 20,000∼25,000 genes [1], alternative splicing allows a single gene to produce multiple transcripts and subsequently multiple proteins that greatly increases protein diversity and their functions [2]. In addition, more than 90% of genes are shown to undergo alternative splicing [3], [4], and many disease-causing mutations introduce alternative mRNA transcripts [5]. It is, therefore, of great importance to effectively measure the levels of gene isoforms in human health and diseases. High-throughput RNA sequencing (RNA-Seq) provides unparalleled dynamic ranges and specificity for transcriptome analysis, while its sample throughput and cost are being improved continuously. An emerging approach for large-scale clinical studies is, therefore, to first sequence with a sufficient depth to comprehensively identify the mRNA transcriptome of the disease, followed by the design of customized microarrays targeting these transcripts as well as by high-throughput screening of thousands of patient samples using the arrays [6].

In addition to providing essential references for array design, the discovery and annotation of new transcripts are also critical to the analysis of both array and RNA-Seq data. The successful deconvolution of isoform expression levels of a gene from microarray data requires a ‘complete’ exon structure not missing any major isoforms of the gene [7]. Similarly statistical inference of isoform expression and their changes from RNA-Seq data also relies on prior annotations of gene transcripts [8]. Currently, the UCSC genome browser includes annotations of human mRNA transcripts from RefSeq [9], UCSC Known Genes [10], and Ensembl [11] (35,971, 77,614, and 143,123 respectively as of June 21, 2010). While each database uses different but overlapping criteria to curate existing sequencing evidences, many new gene isoforms remain to be discovered and catalogued from high-throughput sequencing data.

While generating long-read expressed sequence tags (ESTs) using Sanger sequencing was traditionally the major experimental approach to discover new isoforms of mRNA transcripts [12], the second generation sequencing technologies produce many millions of short reads from mRNA, making it possible to comprehensively analyze the entire transcriptome [13]. Data sets of RNA-Seq have been rapidly accumulated for multiple mammalian tissues [4], [14], various cancers [15], and individual patients [16], which brings the opportunity to discover new mRNA transcripts, i.e. gene isoforms, to a genome-wide scale.

However, currently the reads generated by either Illumina or ABI platforms are typically 50∼100 bases long, much shorter than the full length of mRNA transcripts. For example, a survey of the NCBI Sequence Read Archive (Dec. 1, 2011) indicates that among 4,653 RNA-Seq experiments 84% have read lengths shorter than 100 bp and 76% are single-end reads [17]. In addition, gene transcripts are often not fully covered by uniquely mapped reads of RNA-Seq data because of either the low abundance of the transcripts, the low amplification efficiency of the transcript regions in sample preparation, the insufficient depth of typical sequencing runs, or the high sequence homology. Therefore, the discovery of new transcript isoforms from RNA-Seq data becomes a bioinformatic problem: how to reconstruct a full-length mRNA transcript from short reads by inferring exons and exon-exon junctions that are incompletely covered or completely missing in the sequencing.

The limitations of read length and coverage completeness make it difficult to apply conventional assembly or reconstruction methods to RNA-Seq data. Many short read assemblers for second generation sequencing data construct contigs by extending consensus of overlapping sequencing reads [18], [19], [20], [21]. However, the incomplete coverage of RNA-Seq data significantly limits the length of the reconstructed transcripts, and these short read assemblers commonly target to build sequence contigs rather than full-length transcripts. Other constructors such as ExonWalk [22], [23], which was extensively utilized in the design of exon and junction arrays [6], [7], build full-length transcripts by walking through exons, which is more suitable for long sequencing reads such as ESTs that completely cover several consecutive exons of the transcripts.

While the objective survey of transcriptome by RNA-Seq provides valuable and yet incomplete information on previously unknown transcripts, prior knowledge on annotated transcripts from public databases can compensate the missing information in RNA-Seq data. For example, if sequencing reads alone only identify one of the two junctions of an exon, the other one can be defined by previously known exons sharing the same junction.

In this paper, we propose a computational approach using previously developed SpliceMap [24] as well as ExonMap and JunctionWalk algorithms proposed here to reconstruct new transcripts from RNA-Seq data and prior knowledge on annotated transcripts from existing databases. The proposed method was demonstrated on an RNA-Seq data set of 203 million sequencing reads of 58 bases from human reference muscle tissue [6]. Using strict filtering criteria to control false positives, we identified 2,973 new junctions, 7,471 new exons, and 7,571 new transcripts that were not annotated in RefSeq. These new findings can be included in the future array design and the analysis of alternative splicing in large-scale transcriptome studies [6].

## Results

### Coverage of the RNA-Seq data

203 million RNA-Seq reads from human muscle tissue were mapped over annotated exon and junction regions collected from RefSeq, Ensembl, UCSC Known Genes and EST databases, and 120 million reads were uniquely mapped by allowing up to 2 mismatches. Among the detected exons and junctions with at least one read in the data, 41% of exons and 62% of junctions were covered by fewer than 20 reads because they were from lowly expressed transcripts or in the repeated regions where sequencing reads cannot be mapped with confidence. In addition, for 20% of the detected exons, sequencing data only covered less than 50% of their genomic regions, which made it difficult to recover whole exon regions from the data alone. The overall mapping results show that using RNA-Seq data exclusively is insufficient to build full-length transcripts and additional information on transcripts is required for successful reconstruction.

### Detection of un-annotated junctions and exons

First, junctions were detected from the 203 million mRNA sequencing reads using SpliceMap [24]. In brief, for a given sequencing read, SpliceMap first finds the genomic region where 25 bases at one end of the read can be mapped, and locates the exon-exon junction site by extending the mapping as far as possible toward the other end of the read. Then, it locates the other side of the junction by searching for the rest of the read in the nearby genomic regions.

In total, 203,531 candidate junctions were identified from the data. Comparing with a collection of 200,902 known junctions derived from RefSeq annotations, 130,104 (63%) junctions were already known and 73,427 (37%) were not annotated in the database (Table 1). The 130,104 known junctions cover 65% (130,104/200,902) of all the annotated junctions in RefSeq, showing a good agreement between the sequencing data and the prior knowledge. Transcripts expressed at very low levels or not expressed at all in the muscle tissue, as well as those potentially failed to be amplified during the sample preparation step may have constituted the other 35% of RefSeq junctions.

**Table 1:** Number of observed junctions from the RNA-Seq data and new findings not annotated in RefSeq.

Num. reads supporting the junction	Observed junctions	Un-annotated junctions
1	55,347	43,066
2∼4	38,191	19,442
5∼9	22,792	5,252
10∼19	22,929	2,694
20∼49	27,555	1,791
50∼99	15,633	636
100∼499	16,793	477
500+	4,291	69
Total	203,531	73,427

Next, we sought to identify previously un-annotated exons. An internal exon can be precisely defined by the two junctions that connect it with its adjacent exons. Using the junctions identified from the data by SpliceMap as well as the annotated exon-exon junctions from the reference database, ExonMap detects exons as genomic regions between two junctions if the distance between the two junctions is shorter than a pre-defined threshold, which corresponds to the maximum length of an exon allowed. Here, a length of 10,000 bases was used as the maximum length of an exon because more than 99.97% of exons in RefSeq annotation are shorter than 10,000 bases. Since the first and last exons of transcripts cannot be fully defined by exon-exon junctions, the known start and end positions of transcripts in the reference database were also used together with the junction information to define these exons. For example, an exon can be defined with the start position of a known transcript and a previously un-annotated junction newly identified from the sequencing data. Using RefSeq as a reference database, 107,697 candidate exons were identified by ExonMap, and 12,894 (12%) of these exons were new findings that differed from the annotated exons in RefSeq by at least four bases (Table 2).

**Table 2:** Number of observed exons from the RNA-Seq data and new findings not annotated in RefSeq.

Num. reads supporting the exon	Observed exons	Un-annotated exons
0	18,427	3,224
1	2,568	227
2∼4	6,471	514
5∼9	8,788	634
10∼19	12,092	824
20∼49	17,301	1,297
50∼99	11,450	1,093
100∼499	20,236	2,408
500+	10,364	2,673
Total	107,697	12,894

As expected, the number of detected candidate junctions and exons decreased with the increasing number of sequencing reads mapped to the junctions and exons, and the number of newly identified, previously un-annotated junctions and exons dropped even more rapidly. For example, while 78% of junctions detected by only one sequencing read were previously un-annotated, only 2% of junctions detected by more than 500 reads were new findings. Since SpliceMap reported a 7% false discovery rate for newly found junctions with at least 2 reads and there were likely more false positives among junctions supported by only few reads [24], we chose a cutoff of 20 reads to ensure the presence of the junctions, which is the same criterion used in Wang, *et al.* to detect alternative splicing events [4]. Using this criterion, 2,973 junctions were discovered as new junctions that were not found in RefSeq transcript annotations. Similarly, to ensure high confidence on the newly identified exons, a minimum of 20 reads was required to be mapped to each of these exon regions. With this criterion, 7,471 new exons were identified that were not previously annotated in RefSeq.

**Figure 1:**
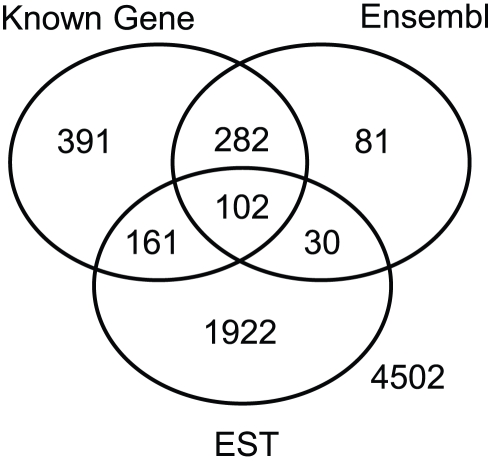
Coverage of the 7,471 new exons identified from RNA-Seq data in other databases. ExonMap was applied to the RNA-Seq data using RefSeq as a reference. 7,471 new exons were identified which were not annotated in RefSeq, among which 4,502 were not annotated in UCSC Known Genes, Ensembl, and EST databases.

We compared these new exons with annotations in other databases that are more comprehensive and include contents with perhaps less experimental support. Figure 1 shows the coverage of these new exons in UCSC Known Genes, Ensembl and EST transcript databases. While 14% of the 7,471 new exons were found in either UCSC Known Genes or Ensembl, 26% of the exons were found only in the EST database, which is expected because the EST database is more comprehensive than the other two annotations. 4,502 (58%) exons were not annotated in any of the three databases. However, since each of these exons were supported by more than 20 reads in the RNA-Seq data, they are very unlikely to be false positives.

### Reconstruction of new transcripts by JunctionWalk

**Figure 2:**
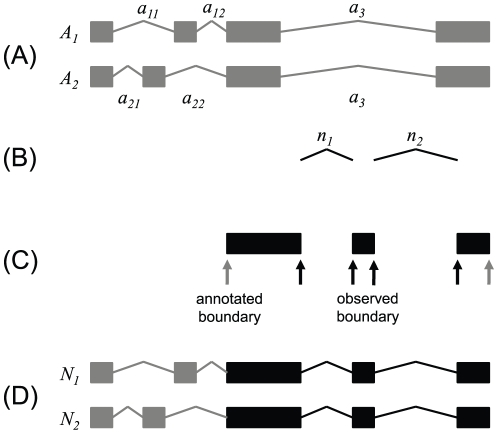
JunctionWalk algorithm. Showing as an example is the application of JunctionWalk algorithm to (**A**) two previously annotated transcripts in the reference database, (**B**) two new junctions identified from RNA-Seq data, and (**C**) three new exons discovered from RNA-Seq data by annotated boundaries in the reference database (gray arrows) or observed boundaries defined by the new junctions from RNA-Seq data (black arrows). A box represents an exon, and a line linking two exons is a junction. From the reference database, transcript *A_1_* has three annotated junctions of *a_11_*, *a_12_*, and *a_3_*, and transcript *A_2_* has *a_21_*, *a_22_* and *a_3_*. From RNA-Seq data, previously un-annotated new junctions of *n_1_* and *n_2_* are defined. The previously annotated exons and junctions are presented in gray, and new ones are in black. (**D**) The algorithm reconstructs new transcript *N_1_* and *N_2_* by walking over the annotated and new exons and junctions.

Finally, we introduce JunctionWalk algorithm to predict full-length mRNA transcripts (Figure 2). Given a set of previously annotated transcripts from the reference database (Figure 2A), newly identified junctions with high confidence by SpliceMap (Figure 2B), and new exons identified with high confidence by ExonMap (Figure 2C), JunctionWalk reconstructs new transcripts (Figure 2D) by walking through the provided exons and junctions. As an example, newly identified exons in Figure 2C are bounded by new junction *n_1_* and *n_2_* identified from sequencing reads, and previously annotated junction *a_12_* and *a_22_* defined from previously known transcripts. To form a complete transcript, new exons and junctions are connected together, and complemented by annotated ones that fill in the missing coverage for transcripts.

This algorithm was applied to our RNA-Seq data of human muscle tissue using RefSeq or Ensembl as a reference transcript database, and identified a significant number of un-annotated transcripts (Table 3). By referring to RefSeq, we identified 7,571 previously un-annotated transcripts in 1,337 RefSeq genes. On average, these newly identified transcripts have a median length of 1,553 bases, and consist of 11 exons and 10 junctions, which include 3 un-annotated exons and 2 un-annotated junctions. The number of new transcripts decreased exponentially with the increasing number of RNA-Seq reads required for the detection of previously un-annotated exons and junctions. Similarly, the reconstruction with Ensembl annotations as the reference predicted 8,980 new transcripts (Table 3) because Ensembl includes a larger number of annotated transcripts than RefSeq.

**Table 3:** Number of previously un-annotated transcripts reconstructed using RefSeq or Ensembl annotations as the reference.

Num. reads supporting the transcript	RefSeq	Ensembl
≥10	30,005	33,514
≥20	7,571	8,980
≥50	1,616	2,023
≥100	734	1,020

**Figure 3:**
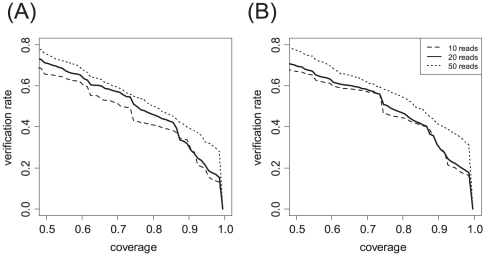
Verification rates in EST of new transcripts reconstructed from the data. The transcripts were reconstructed by using (**A**) RefSeq and (**B**) Ensembl as the reference databases. The solid line is the verification rate by transcript sequences in the EST database for the constructed transcripts supported by with more than 20 reads, the dashed line is with more than 10 reads, and the dotted line is with more than 50 sequencing reads.

To evaluate the performance of the reconstruction algorithm, we compared the transcripts computationally reconstructed from the RNA-Seq data and using either RefSeq and Ensembl as prior knowledge with transcript sequences in the EST database. Only transcripts un-annotated in the prior knowledge used for the reconstruction were compared with the EST database. Since a large number of ESTs in the EST database are not full-length transcripts, we compared each reconstructed transcript with each of the ESTs by calculating the percentage of nucleotide bases overlapped between the two sequences. As shown in Figure 3A (the solid line), 73% of the 7,571 new transcripts reconstructed from RNA-Seq data and RefSeq reference were verified by ESTs with more than 50% coverage. Similarly, 75% of the 8,980 new transcripts from RNA-Seq and Ensembl reference were verified with >50% EST coverage (Figure 3B, the solid line). Further, new transcripts constructed from new exons and junctions each supported by at least 50 reads (the dotted lines) have higher percentages of EST coverage than from those supported by 20 reads (the solid lines) or 10 reads (the dashed lines), suggesting that reconstructed transcripts supported by a larger number of RNA-Seq reads likely have higher confidence.

**Figure 4:**
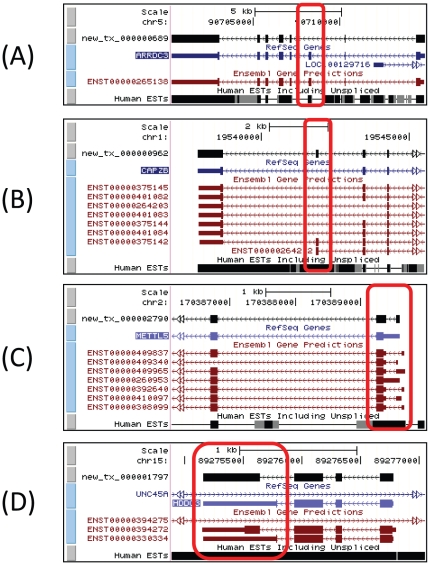
Types of alternative splicing events found in the newly reconstructed transcripts. These transcripts differ from the annotated transcripts of the same genes in RefSeq by (**A**) skipping a known exon, (**B**) inserting a new exon, (**C**) splitting a known exon, and (**D**) having a different end of a known exon. The first transcript track in black on top of each panel presents a reconstructed transcript, the second track in blue presents the annotated transcript in RefSeq which was used as the reference for the reconstruction, the third set of tracks in brown represent transcripts of the same gene in Ensembl, and the track in black at the bottom is a ‘dense’ presentation of the ESTs, because of the large number of ESTs.

Examination of the structures of the new transcripts reconstructed from RNA-Seq data and RefSeq reference reveals that they differ from the previously annotated transcripts in RefSeq by skipping previously known exons, adding previously unknown exons or introns, or including exons with alternative starts or ends. As examples, Figure 4A shows a new transcript skipping a previously annotated exon in RefSeq, which was discovered by the identification of a previously unknown junction bridging the two neighboring exons of the skipped exon. Similarly, Figure 4B shows a new transcript consisting of a previously un-annotated exon, which was defined by two new junctions bridging two previously known exons on each side to the un-annotated exon. Figure 4C shows a previously annotated exon split into two new exons with the introduction of an intron, which was discovered by two new junctions within the annotated exon. Figure 4D presents a reconstructed transcript with an exon that has an alternative end, where the leftmost exon with the alternative end was connected to the second leftmost exon by a new junction in the RNA-Seq data. The reconstructed transcripts in Figure 4B, C and D are supported by Ensembl while the transcript in Figure 4A is not annotated in either RefSeq or Ensembl. These examples show that the approach of reconstruction is capable of capturing different alternative structures.

Among the 7,571 reconstructed transcripts not previously annotated in RefSeq, 2,167 (29%) transcripts have less than 50% coverage with EST annotations, which are subject to further evaluations using experimental data. However, since each exon and junction of these new transcripts is either well supported by RNA-Seq data (of more than 20 reads) or from prior annotations in RefSeq, these are also new candidates to be included in the design of custom arrays and in the future transcriptome research.

## Discussion

In this work, we proposed a knowledge-based approach to reconstruct new mRNA transcripts from short sequencing reads. By utilizing previously annotated exons and junctions to fill in the missing information of experimental data of RNA-Seq, we showed that the proposed algorithm is able to construct long transcripts from short reads. As a comparison, we applied two *de novo* assembly methods to our RNA-Seq data that utilize no existing knowledge of annotated transcripts. Velvet, an assembly algorithm based on de Bruijin graphs [18], reconstructed transcripts with a median length of 207 bases, and Trinity, another *de novo* algorithm that does not rely on aligning reads to a reference genome, reported a median length of transcripts of 173 bases [25], which are much shorter than the median length 1,553 bases from our algorithm. These results corroborate the usefulness of leveraging prior gene annotations in the reconstruction of mRNA transcripts from sequencing data.

Very recently, Roberts *et al.* described an interesting alternative approach of reference annotation based transcript (RABT) assembly, where faux reads were first generated to tile every reference transcript, and the Cufflinks assembler was then applied to compute the minimum number of transfrags that explain both sequencing data and the faux reads [26]. While this approach is useful for RNA-Seq data analysis, the parsimonious assembly constructs a new exon to only one of the reference transcripts that share the same exon-exon junctions. To illustrate this, Figure S1 shows a simplified example of two reference transcripts (of TPM2 gene) and sequencing reads tiling across a new exon (highlighted) and its connected exons. While RABT assembled the new exon with one of the two reference transcripts, our method discovered both new transcripts. Since most of the human genes have more than one annotated transcript isoforms that share exon-exon junctions, we found that the assembled transfrags from the current RABT are problematic when applied to array design and analysis. In addition, in contrast to the pure assembler approach, our method explicitly defines and utilizes exons, exon boundaries, and exon-exon junctions, which can be directly applied to the design and analysis of transcriptome arrays [6].

While here we utilized a sequencing data set of 58 bps as a demonstration example, our algorithm is expected to be applicable to RNA-Seq data with longer read length and paired-end data as well. Our proposed approach can be further improved by utilizing additional information such as sequence conservation and transcript structures [27], [28]. As the sequencing data sets from increasing number of tissues, physiological states and diseases are becoming available, the confidence on a novel reconstructed transcript is increased if it has been identified in multiple data sets. Besides, the boundaries of the starting and ending exons of the reconstructed transcripts can also be improved by the accumulation of deeper sequencing data as well as the estimation of the poly-A tail cleavage sites [29]. Finally, further developments are required to combining the strength of *de novo* assemblers with the knowledge-based reconstructions.

In summary, we demonstrated that the limitations of the short read length of RNA-Seq and its incomplete coverage of full-length gene transcripts can be partially overcome by utilizing prior transcript annotations from reference databases. This algorithm has been utilized by the NIGMS *Inflammation and the Host Response to Injury* Glue Grant program in the design and revision of the human transcriptome array for large-scale clinical studies [6]. With the continuing technical improvements of sequencing technologies, especially on the sequencing cost and sample throughput, RNA-Seq data of human transcriptome under various biological conditions will likely be accumulated exponentially in the near future, which provides an unprecedented opportunity to systematically discover new transcripts of human genome. The resulting comprehensive catalogue of human gene transcripts will provide an essential reference for transcriptome studies, including the design and revision of customized exon-junction arrays for large-scale clinical studies [6] as well as the computational analysis of microarray and RNA-Seq data for genome-wide alternative splicing in biological and clinical studies [7].

## Methods

### mRNA sequencing

mRNA was purified from total human muscle RNA, processed and sequenced using the Illumina Genome Analyzer following protocols recommended by the manufacturer. From two sequencing runs, 203 million reads with 58 bases were acquired [6]. Sequencing reads were mapped over the exon and junction regions of RefSeq using SeqMap with 2 mismatches allowed [30]. Among the 203 million total reads, 128,916,392 (63.6%) reads were mapped over exon and junction regions, and 119,576,008 (59.0%) were uniquely mapped. These percentages are comparable to previously reported results [31].

### ExonMap algorithm

SpliceMap performs junction discovery using RNA-Seq data, based on sequence mapping and splicing signals [24]. As an input, ExonMap takes these newly identified junctions that are not annotated previously in databases as well as a set of annotated exons and exon-exon junctions. It searches both sides of a newly identified junction for either another newly identified junction or a known junction within a defined search window. ExonMap then defines one observed exon between the two junctions. In this study, a collection of 221,022 annotated exons and 200,902 junctions from 35,971 transcripts in RefSeq (Release 41) was used as the reference, and a window of 10,000 bases was used as the searching window.

### JunctionWalk algorithm

JunctionWalk algorithm reconstructs candidate full-length transcripts from a set of previously un-annotated and annotated exons and junctions. Previously annotated exons and junctions are derived from a reference transcript database such as RefSeq and Ensembl. The previously annotated junctions are defined as junctions observed between two adjacent exons in a transcript in the reference database. The un-annotated junctions are not included in the collection of annotated junctions but identified from RNA-Seq data by SpliceMap. Similarly, the un-annotated exons are newly derived exons by ExonMap algorithm and not previously annotated.

To reconstruct transcripts of a gene, two junctions are assigned to the same transcript if and only if the following conditions are satisfied:

1. The two junctions are on the same strand of a chromosome.
2. The two junctions do not overlap in genome coordinates.
3. The exon defined by the two junctions belongs to the set of un-annotated or previously annotated exons.
4. The exon in condition 3 is not longer than a pre-defined gene specific threshold.
5. If the two junctions are both previously annotated, they are observed together on at least one transcript in the reference database.

Conditions 1 and 2 are obvious. Condition 3 guarantees that every exon of a reconstructed transcript is either known or identified from the sequencing data. Condition 4 is to avoid generating artificially long exons. In this study, the gene specific threshold for condition 4 is defined as the larger value between 1,000 bases and the maximum length of annotated exons of the corresponding gene. Since 95% of exons in RefSeq are shorter than 1,000 bases, this gene specific threshold provides a reasonable upper bound for the length of the potential exon of each gene. Condition 5 prevents junctions that belong exclusively to different transcripts to be assigned together to the same transcript without experimental evidence. JunctionWalk algorithm reconstructs potentially full-length transcripts by connecting all junctions that can be put together.

Starting from a junction, this algorithm extends a transcript by walking over junctions in the order of their genomic positions. First, a starting junction is selected. The first junction of any annotated transcript in the reference database can be a starting junction. If there is an un-annotated junction identified from RNA-Seq data before the first annotated junction on genome coordinates, the un-annotated junction also can be a starting junction. Then, the algorithm walks to the next junction if the second junction can be put together with the previous junction according to the five conditions listed above. By continuing walking until there are no more junctions left that meet the criteria, JunctionWalk algorithm completes the reconstruction of a transcript. If there are multiple junctions that can be walked over in the middle of the process, each branch of the walking will be processed resulting the reconstruction of different transcripts.

Figure 2 illustrates how JunctionWalk reconstructs a new transcript. For a gene with two annotated transcripts (Figure 2A), assume that two un-annotated junctions (Figure 2B) and three un-annotated exons (Figure 2C) are newly discovered from sequencing data. Both junction *a_11_* and *a_21_* can be a starting junction of the walking process. Starting from junction *a_11_*, the algorithm moves to junction *a_12_*. Since the pre-defined maximum exon length in condition 4 is longer than or equal to the maximum length of annotated exons, two annotated junctions can always be put together if they are from the same annotated transcript. From junction *a_12_*, it can walk to both junction *a_3_* and junction *n_1_*. The reconstruction moving to junction *a_3_* ends up to be annotated transcript *A_1_*. The walking over junction *n_1_* continues to junction *n_2_*, and finally reconstructs new transcript *N_1_* (Figure 2D). Similarly, transcript *N_2_* is constructed by a walking process starting from junction *a_21_*. Exons on reconstructed transcripts are either known or newly identified by ExonMap with two neighboring junctions (Figure 2C). Exons at each end of a transcript are defined by the nearest annotated exon boundary. The ending exons of the reconstructed transcripts in Figure 2D are defined with the boundary of the first and last exons of the annotated transcripts in Figure 2A.

## Supporting Information

Figure S1 **A reconstruction example of TPM2.** Our proposed algorithm was compared with the Cufflink reference annotation based transcript (RABT) assembly algorithm [Robert *et al*, Bioinformatics, 2011] over a simplified example of TPM2 gene. Given two reference transcripts of the gene (Ref1 and 2), sequencing reads tiling across a new exon (highlighted) and its connected exons were fed into each algorithm. The Cufflink RABT was able to assemble only one new transcript where the new exon is assembled with one of the two reference isoforms of the gene (CUFF.2.2) while our proposed algorithm generated two new transcripts with the new exon integrated to each of the two reference isoforms (JucWalk 1 and 2).
(EPS)

## References

[1] International Human Genome Consortium (2004). Finishing the euchromatic sequence of the human genome.. Nature.

[2] Matlin AJ, Clark F, Smith CW (2005). Understanding alternative splicing: towards a cellular code.. Nat Rev Mol Cell Biol.

[3] Kampa D, Cheng J, Kapranov P, Yamanaka M, Brubaker S (2004). Novel RNAs identified from an in-depth analysis of the transcriptome of human chromosomes 21 and 22.. Genome Res.

[4] Wang ET, Sandberg R, Luo S, Khrebtukova I, Zhang L (2008). Alternative isoform regulation in human tissue transcriptomes.. Nature.

[5] Wang GS, Cooper TA (2007). Splicing in disease: disruption of the splicing code and the decoding machinery.. Nat Rev Genet.

[6] Xu W, Seok J, Mindrinos MN, Schweitzer AC, Jiang H (2011). Human transcriptome array for high-throughput clinical studies.. Proc Natl Acad Sci U S A.

[7] Hiller D, Jiang H, Xu W, Wong WH (2009). Identifiability of isoform deconvolution from junction arrays and RNA-Seq.. Bioinformatics.

[8] Jiang H, Wong WH (2009). Statistical inferences for isoform expression in RNA-Seq.. Bioinformatics.

[9] Pruitt KD, Tatusova T, Maglott DR (2007). NCBI reference sequences (RefSeq): a curated non-redundant sequence database of genomes, transcripts and proteins.. Nucleic Acids Res.

[10] Hsu F, Kent WJ, Clawson H, Kuhn RM, Diekhans M (2006). The UCSC Known Genes.. Bioinformatics.

[11] Flicek P, Amode MR, Barrell D, Beal K, Brent S (2011). Ensembl 2011.. Nucleic acids research.

[12] Adams MD, Kelley JM, Gocayne JD, Dubnick M, Polymeropoulos MH (1991). Complementary DNA sequencing: expressed sequence tags and human genome project.. Science.

[13] Wang Z, Gerstein M, Snyder M (2009). RNA-Seq: a revolutionary tool for transcriptomics.. Nat Rev Genet.

[14] Mortazavi A, Williams BA, McCue K, Schaeffer L, Wold B (2008). Mapping and quantifying mammalian transcriptomes by RNA-Seq.. Nat Methods.

[15] Levin JZ, Berger MF, Adiconis X, Rogov P, Melnikov A (2009). Targeted next-generation sequencing of a cancer transcriptome enhances detection of sequence variants and novel fusion transcripts.. Genome Biol.

[16] Pflueger D, Terry S, Sboner A, Habegger L, Esgueva R (2010). Discovery of non-ETS gene fusions in human prostate cancer using next-generation RNA sequencing.. Genome Res.

[17] Leinonen R, Sugawara H, Shumway M (2011). The sequence read archive.. Nucleic acids research.

[18] Zerbino DR, Birney E (2008). Velvet: algorithms for de novo short read assembly using de Bruijn graphs.. Genome Res.

[19] Simpson JT, Wong K, Jackman SD, Schein JE, Jones SJ (2009). ABySS: a parallel assembler for short read sequence data.. Genome Res.

[20] Butler J, MacCallum I, Kleber M, Shlyakhter IA, Belmonte MK (2008). ALLPATHS: de novo assembly of whole-genome shotgun microreads.. Genome Res.

[21] Bryant DW, Wong WK, Mockler TC (2009). QSRA: a quality-value guided de novo short read assembler.. BMC Bioinformatics.

[22] Karolchik D, Baertsch R, Diekhans M, Furey TS, Hinrichs A (2003). The UCSC Genome Browser Database.. Nucleic Acids Res.

[23] Sugnet CW, Kent WJ, Ares M, Haussler D (2004). Transcriptome and genome conservation of alternative splicing events in humans and mice.. Pac Symp Biocomput.

[24] Au KF, Jiang H, Lin L, Xing Y, Wong WH (2010). Detection of splice junctions from paired-end RNA-seq data by SpliceMap.. Nucleic Acids Res.

[25] Grabherr MG, Haas BJ, Yassour M, Levin JZ, Thompson DA (2011). Full-length transcriptome assembly from RNA-Seq data without a reference genome.. Nature biotechnology.

[26] Roberts A, Pimentel H, Trapnell C, Pachter L (2011). Identification of novel transcripts in annotated genomes using RNA-Seq.. Bioinformatics.

[27] Thomas JW, Touchman JW, Blakesley RW, Bouffard GG, Beckstrom-Sternberg SM (2003). Comparative analyses of multi-species sequences from targeted genomic regions.. Nature.

[28] Burge C, Karlin S (1997). Prediction of complete gene structures in human genomic DNA.. Journal of molecular biology.

[29] Pickrell JK, Marioni JC, Pai AA, Degner JF, Engelhardt BE (2010). Understanding mechanisms underlying human gene expression variation with RNA sequencing.. Nature.

[30] Jiang H, Wong WH (2008). SeqMap: mapping massive amount of oligonucleotides to the genome.. Bioinformatics.

[31] Bullard JH, Purdom EA, Hansen KD, Durinck S, Dudoit S (2009). Statistical Inference in mRNA-Seq: Exploratory Data Analysis and Differential Expression..

